# Social participation and attitude towards gerontechnology: The moderating role of frailty status

**DOI:** 10.1093/geroni/igab046.2489

**Published:** 2021-12-17

**Authors:** Ka Yeon Lee, Sun Ah Lee, Susanna Joo, YoonMyung Kim, Yun Mook Lim, Hey Jung Jun

**Affiliations:** 1 Yonsei University, Seoul, Seoul-t'ukpyolsi, Republic of Korea; 2 The Pennsylvania State University, University Park, Pennsylvania, United States; 3 Yonsei University, Seodaemun-gu, Seoul-t'ukpyolsi, Republic of Korea

## Abstract

The purpose of this study was to examine whether frailty status moderates the association between social participation and attitude towards using gerontechnology. The sample was Korean older adults without cognitive impairment (N = 310, aged 66-90, 51% women) who completed an online survey. The attitude towards using gerontechnology was measured with two questions from the Senior Technology Acceptance Model (Chen & Chan, 2014), asking whether using technology is a good idea and whether they like the idea of using technology. Social participation was assessed by asking whether the participants engage in social or community activities on a scale of 1–10. Frailty status was determined based on the Korean Groningen Frailty Indicator (K-GFI). Covariates were age, gender, marital status, employment status, education level, and household income. Results from regression analyses showed significant interaction between frailty status and social participation on attitude towards using gerontechnology. Specifically, social participation was associated with positive attitude towards using gerontechnology among non-frail older adults. This association was not significant among frail older adults. Our findings suggest that the relationship between social participation and attitude towards using gerontechnology might differ by physical health status. Among older adults who are physically healthy and actively participate in social activities, the attitude towards using gerontechnology might be more positive due to greater exposure to new technology-related information. Future studies need to address alternative ways to enhance technology-friendliness among older adults with poor physical health.

